# Fallopian Tube Tumor Mimicking Primary Gastrointestinal Malignancy

**DOI:** 10.7759/cureus.9795

**Published:** 2020-08-17

**Authors:** Anupam K Gupta, Oscar A Vazquez

**Affiliations:** 1 Minimally Invasive Surgery, University of Miami Hospital, Miami, USA; 2 Surgery, Charles E. Schmidt College of Medicine, Florida Atlantic University, Boca Raton, USA

**Keywords:** malignancy, gi bleed, fallopian tube, tumor, carcinoma

## Abstract

A 67-year-old female patient positive for a mismatch repair gene mutation and history of serous carcinoma of the fallopian tube presented with a lower gastrointestinal bleed. Clinical workup was suggestive of a primary gastrointestinal malignancy. Pathological review after right hemicolectomy revealed the primary tumor was a fallopian tube carcinoma. Over the next few years, she presented with upper and lower gastrointestinal bleeds from a recurrent metastatic disease, which was from the primary fallopian tube cancer. Although serous carcinoma of the fallopian tube is not an uncommon diagnosis, it is unusual for it to present with symptoms of recurrent gastrointestinal bleed mimicking a primary gastrointestinal malignancy.

## Introduction

Primary fallopian tube carcinoma (PFTC) is a sporadic gynecologic malignant tumor and accounts for approximately 0.14%-1.8% of female genital malignancies [1-3]. The most common route of dissemination is metastasis through the peritoneal cavity with rare instances of hematogenous spread. Distant metastasis often occurs in the liver, brain, or lung [4-6]. Gynecological malignancies in their advanced stages are known to cause bowel obstruction and, most commonly it will cause bowel involvement with extrinsic compression, adhesion, or carcinomatosis [7]. It is unusual for gynecological malignancy to present with gastrointestinal (GI) bleeding mimicking primary colon cancer as it has been reported very few times in the literature, with one case of metastatic ovarian carcinoma presenting as sigmoid colon malignancy [6]. It is also uncommon for it to present with invasion into the bowel wall with a recurrent GI bleed.

## Case presentation

**Figure 1:**
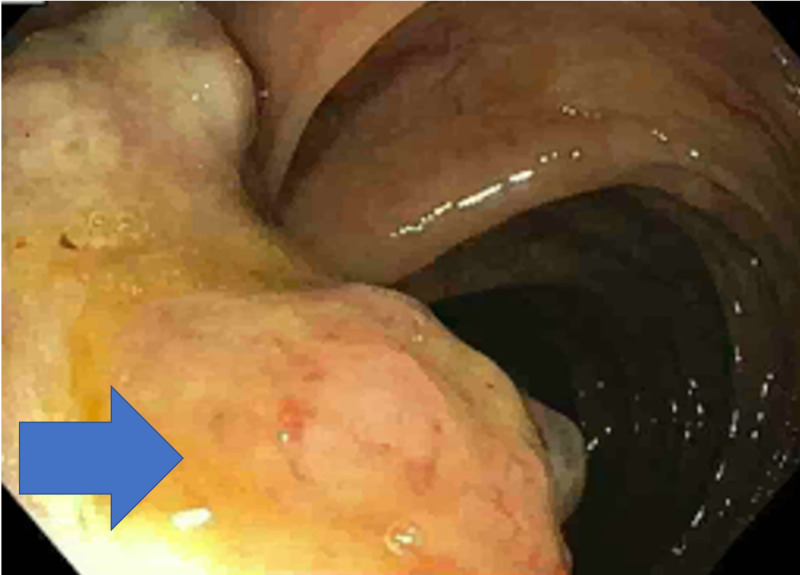
Colonosopy showing ulcerated mass in transverse colon (blue arrow).

**Figure 2:**
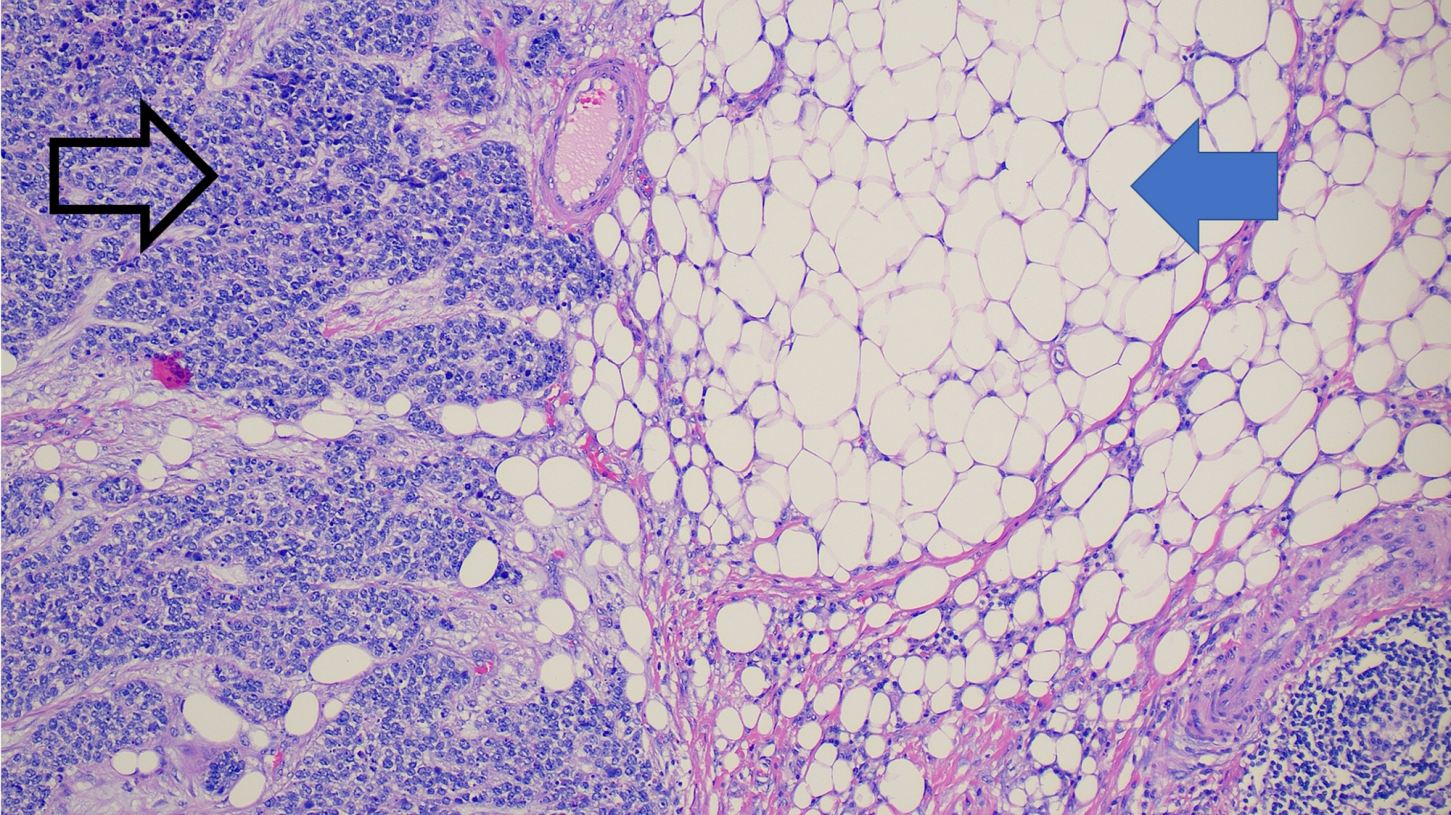
Hematoxilyn and eosin stain showing poorly differentiated carcinoma (black arrow) involving right pericolic adipose tissue (blue arrow).

A 67-year-old female patient with a history of glaucoma and left fallopian tube serous adenocarcinoma status post total abdominal hysterectomy and right salpingo-oophorectomy presented with symptoms of lower GI bleeding and anemia for which she underwent colonoscopy. Her family history was positive for endometrial cancer in her sister, and genetic studies showed mutations in mismatch repair genes suggestive of hereditary non-polyposis colorectal cancer (Lynch syndrome - MLH1/MSH2/MSH6/PMS2). Immunohistochemistry of the fallopian tube tumor was positive for Ber-Ep4, EMA, and WT1. It was negative for calretinin with some cells positive for estrogen receptor (ER) immunostain (consistent with high-grade serous adenocarcinoma of Müllerian origin). Colonoscopy revealed that the patient had a proximal transverse colon mass, which appeared as a primary colon malignant mass (Figure 1). Assuming this was primary colon malignancy, she underwent right hemicolectomy with the intraoperative presence of enlarged lymphatics along the ileocolic trunk. She had elevated CA-125 levels, and carcinoembryonic antigen levels were within normal limits. Pathology showed poorly differentiated carcinoma involving right pericolic adipose tissue (Figure 2) suggestive of primary fallopian cancer origin. The patient was discharged in stable condition and placed on gemcitabine chemotherapy after this for which she completed three out of six cycles.

**Figure 3:**
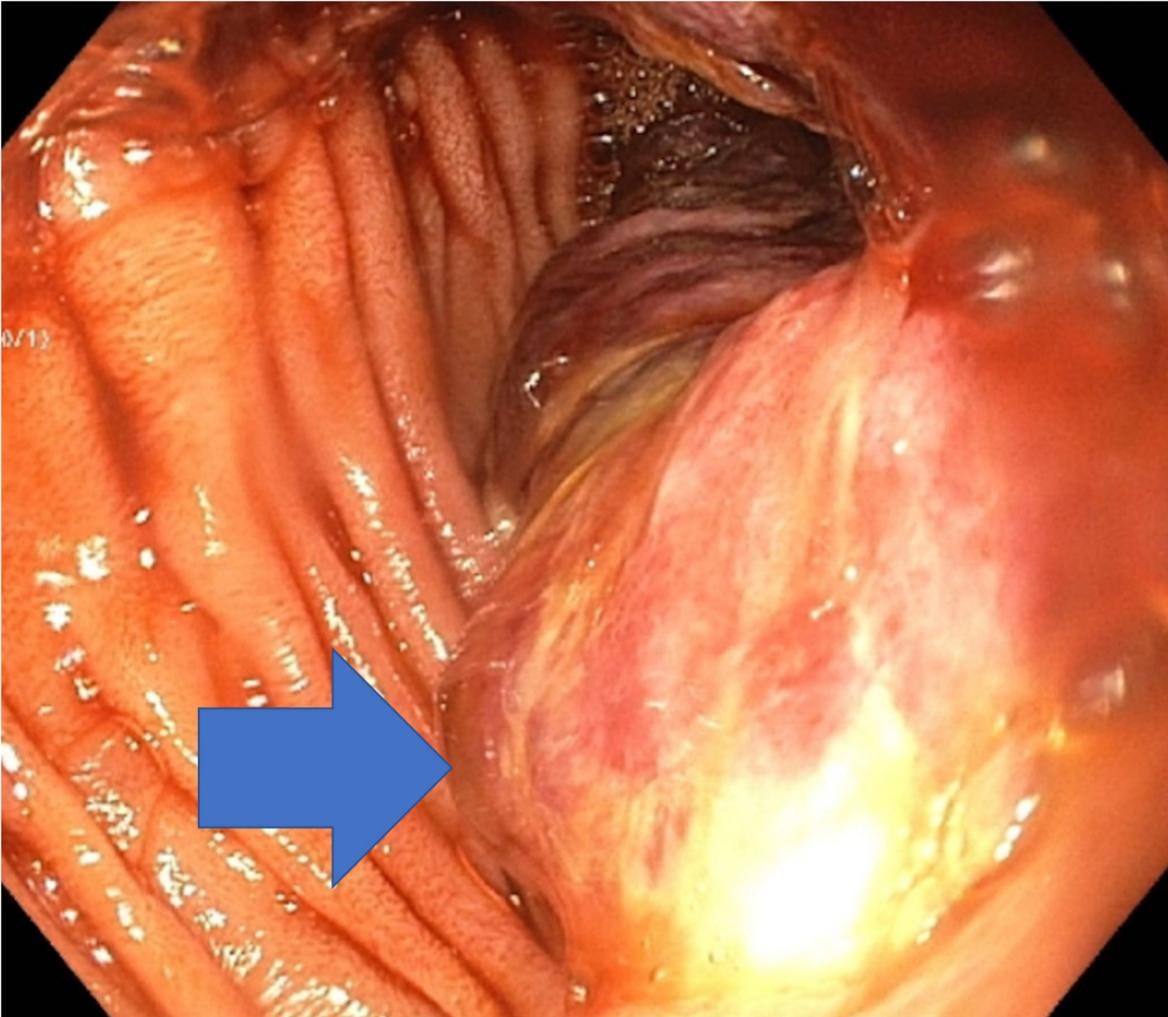
Colonoscopy showing mass at the second part of the duodenum (blue arrow).

**Figure 4:**
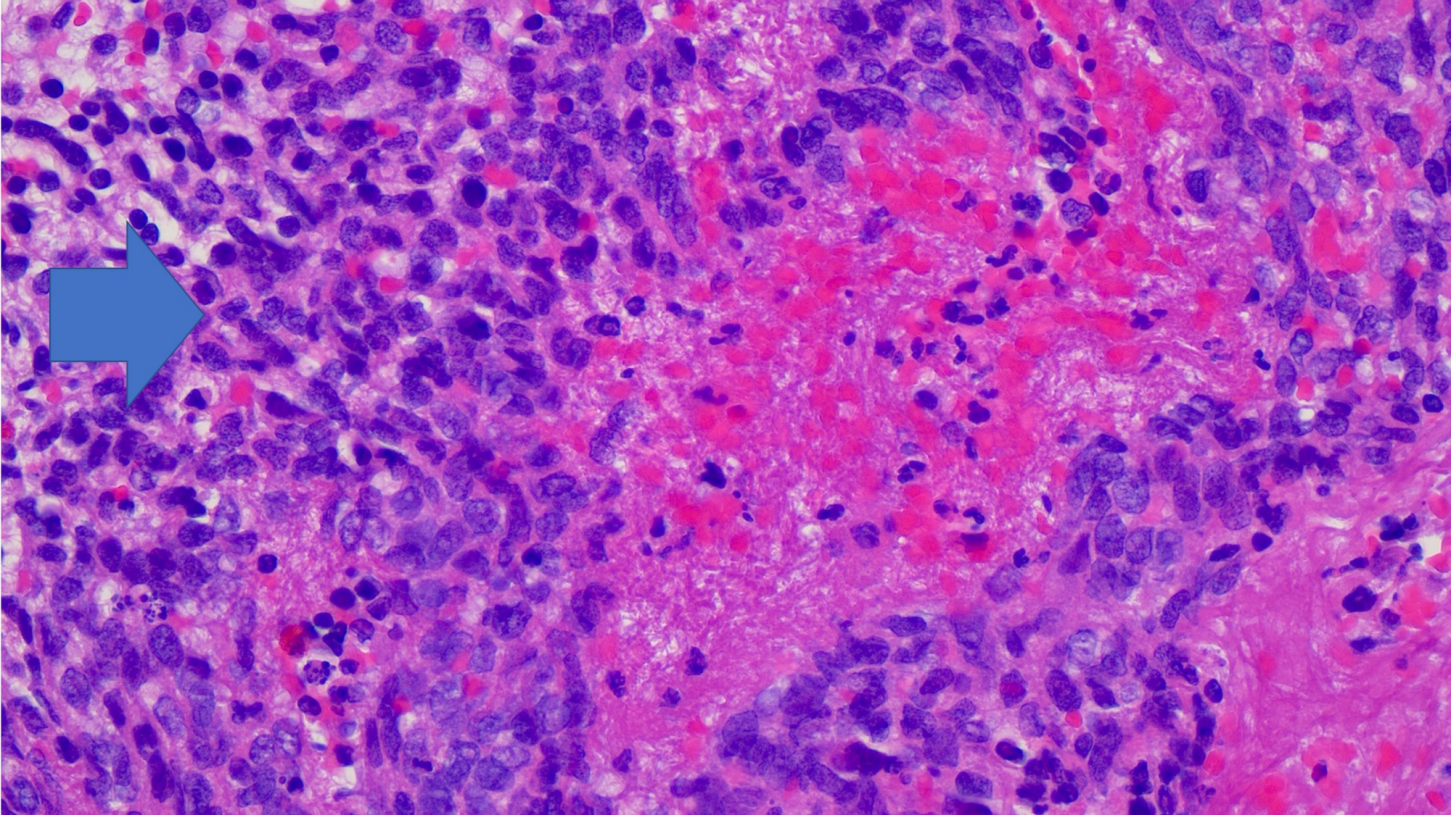
Hematoxylin and eosin stain showing poorly differentiated carcinoma (blue arrow).

**Figure 5:**
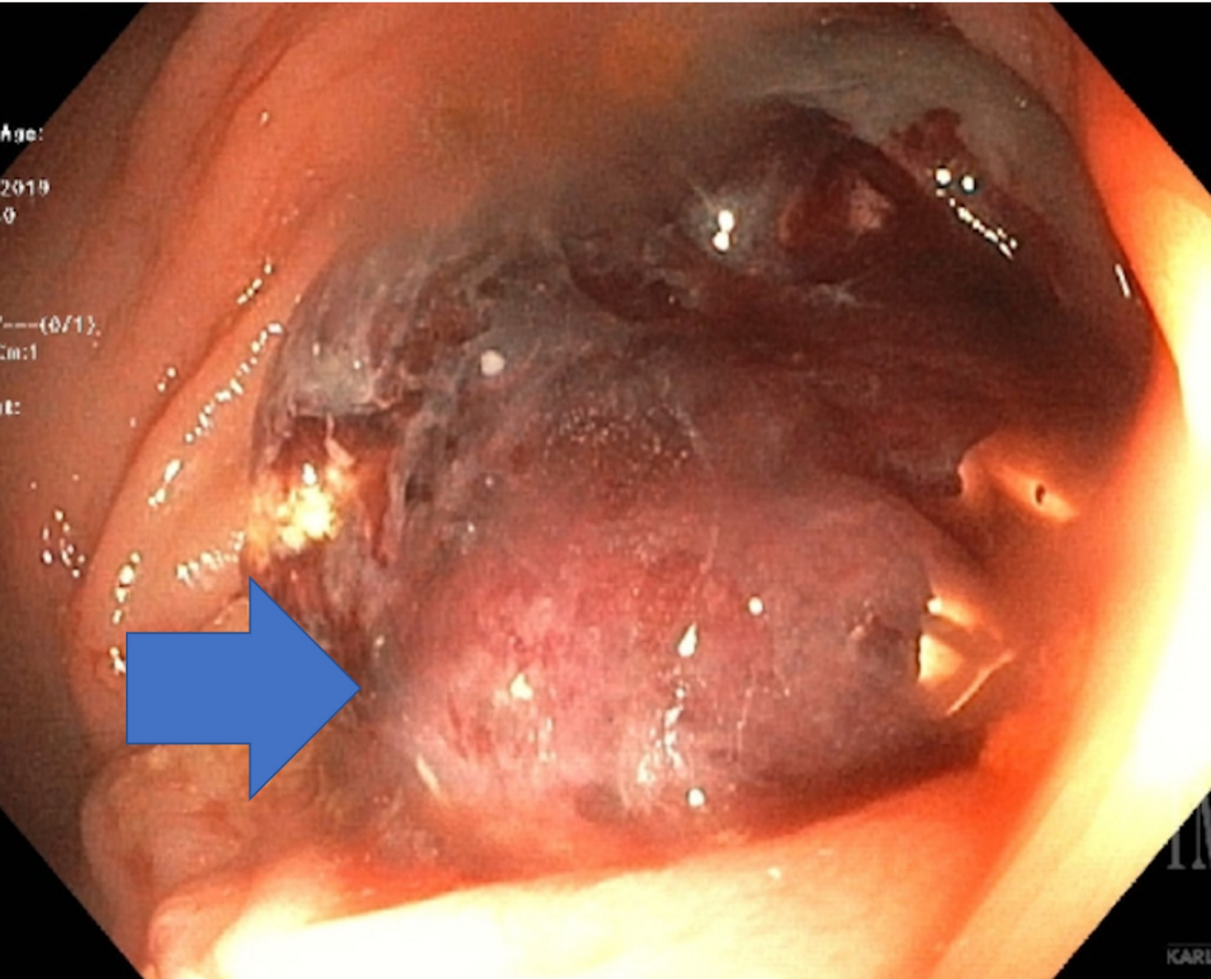
Colonoscopy showing rectal growth of fallopian tube origin (blue arrow).

Two years from the right hemicolectomy, the patient presented with melena, weakness, hypotension, fatigue, and a hemoglobin of 5.3 g/dL. An upper GI endoscopy revealed an ulcerated mass in the second part of the duodenum (Figure 3). Biopsy of the duodenal growth showed poorly differentiated carcinoma (Figure 4). Two months later, the patient presented with cachexia and a lower GI bleed where a colonoscopy revealed a rectal growth with pathology report confirming fallopian tube origin (Figure 5). Ultimately, the patient denied colonoscopy, or any surgical intervention, and she was discharged to a hospice center where she subsequently succumbed to extensive metastatic tumor burden.

## Discussion

PFTC histologically and clinically resembles ovarian epithelial carcinoma and presents within the peritoneum where it stays throughout its course. It can metastasize to other peritoneal surfaces by exfoliating cells that implant throughout the cavity with the intraperitoneal route of dissemination being considered as the most common [8-10]. Its incidence has been rising and varies between 2.9/1,000,000 and 5.7/1,000,000 during the last decades [11]. Up to 40%-60% of high-grade serous carcinomas of the ovary or peritoneum may have fallopian tube fimbriae origin as per genetic, molecular, and histologic evidence [12]. Chronic tubal inflammation, infertility, tuberculous salpingitis, and tubal endometriosis can be associated with PFTC, but the exact etiology is still unknown, though it is associated with BRCA germline and TP53 mutations (similarly to ovarian carcinoma) [13-15].

Alhough PFTC can metastasize to the colon typically, the GI involvement is usually limited to the seromuscular layer of the small and large bowel and its mesentery [16]. However, in our patient, the presentation in the right colon mimicking a primary and histopathology is suggestive of the possible lymphatic route or spread, which is unusual. Kadakia et al. described the most common endoscopic findings of metastatic GI malignancies as ulcerated nodules, ulcerated submucosal masses, umbilicated nodules with central exudate, and necrotic ulcers with heaped-up margins [17]. This morphology is consistent with the duodenal and rectal metastasis found on upper and lower endoscopy later in this patient's course. As mentioned by Park et al., it is essential to identify the primary malignancy early, especially in a patient like ours, with a history of mismatch repair gene mutation and PFTC diagnosis [6]. This is important because it can help guide treatment options since colorectal cancer is usually treated with 5-fluorouracil and platinum agents, as opposed to fallopian tube cancer which is treated with paclitaxel and platinum agents [12,18]

## Conclusions

PFTC is a rare tumor that can metastasize by an intraperitoneal route. It is unusual for it to present with recurrent GI bleeding mimicking a primary GI malignancy. Management is guided by identifying tumor origin based on history of other primary malignancies and they should be ruled out, even in a single intraluminal lesion.
